# Development and evaluating the biopotency of ready to eat liver meat balls in fighting anaemia and vitamin A deficiency, improving selected nutritional biochemical indicators and promoting the cognitive function among mildly anaemic Egyptian children aged 3–9 years

**DOI:** 10.1017/S1368980022000970

**Published:** 2022-11

**Authors:** Rania Bassouni, Magda Soliman, Laila Abbas Hussein, Zeinab Monir, Ashraf A Abd El-Meged

**Affiliations:** 1 Department of Nutrition and Food Science, National Research Centre, Cairo, Egypt; 2 Department of Child Health, National Research Centre, Cairo, Egypt; 3 Department of Food Science and Nutrition, Helwan University, Cairo, Egypt

**Keywords:** Ready to eat liver meat balls, Egyptian children 3–9 years, Anaemia, Vitamin A deficiency, Systemic Ig A, Urinary hydroxyl proline index, Cognitive function

## Abstract

**Objective:**

Ready to eat fried liver meat balls (LMB) were developed to fight anaemia and vitamin A deficiency and promote cognitive function.

**Design:**

Randomised controlled trial consisting of two arms: control group with no supplement and LMB group receiving LMB supplement three times a week for 90 d. Criteria of evaluations included dietary assessment, anthropometric measurements, laboratory investigations and cognitive function by Wechsler test.

**Setting:**

Kinder Garten and primary school in Urban Giza.

**Participants:**

Sixty boys and girls aging 3–9 years.

**Results:**

The LMB supplement contributed to significant increases in the intakes of high bioavailable Fe and vitamin A in the diets of all children. Initial overall prevalence of mild and moderate anaemia was 43 %, which disappeared completely from all children aging < 72 months and from 88 % of children ≥ 72 months after the 90 d dietary intervention with the LMB. Faecal systemic immune globulin A, urinary hydroxyproline index and urinary iodine excretion increased significantly (*P* < 0·05) only after the dietary intervention with the LMB supplement for 90 d. The standard scores of verbal and non-verbal cognitive function tests (Δ day 90–day 0) increased significantly (*P* < 0·05) among the LMB group compared with the respective changes observed among the control group. The increase in height-for-age Z score and blood Hb were good predictors for improvement in cognitive function.

**Conclusion:**

LMB supplement is effective sustainable nutritious biotherapeutic food in fighting hidden hunger and promoting the cognitive function.

The sustainable development goals 2030 created in the year 2015 by the United Nation organisation imply that each of the 193 member states should do substantial efforts and undertake actions to reduce the stunting, wasting and anaemia, indicators of hunger by 50 % by the year 2030 (SDG, 2030)^(1)^. The economic cost of hidden hunger, represented by Fe-deficiency anaemia, Zn deficiency, vitamin A deficiency and iodine amount to a loss of 2·5% of the Indian gross domestic product^(2)^. The most commonly used strategies to combat Fe deficiency are the provision of Fe supplements as FeSO_4_, which have limited significant impact on BMI^(3–8)^, health and nutritional status among adolescents in low- and middle-income countries^(9)^. Currently, there is general agreement that Fe fortification of low bioavailability diets had limited effect on Fe status^(10,11)^. Meanwhile, the higher food synergy of animal-sourced foods (ASF) render them superior in raising the low bioavailability of dietary Fe relative to the respective technologically reduced Fe-fortified cereal products^(12)^. ASF consumption indicator associated negatively with stunting in studies involving 30 000 children in sixty-one countries from five continents^(13)^. ASF-based supplementation to young children increased weight by ≤ 0·14 kg and the height-for-age Z score by +0·06 and are considered the optimum option for improving nutritional quality of a largely plant-source based diet.^(14)^. Supplementing the meals of African children with cooked beef improved their growth, cognitive and behavioural outcomes^(15–18)^. The intake of animal products is usually constrained by their cost, but the edible internal organs, which makes considerable proportion of the carcass weight of chicken and farm animals are highly nutritious at reasonable prices^(19)^. Chicken liver contains high Fe content, when compared with the Fe levels in beef liver (29·3–66·71 mg/kg)^(78)^. The absence of vitamin A deficiency among South African preschool children is due to the common consumption of 60–75 g of liver per month^(20)^. World’s frozen chicken liver with average price of 540–560 US$ per metric tons (1000 kg) is far much cost effective than the respective price of 143 US$ for one single package of American fortified maize soyabean flour blend, required for the dietary management of an African malnourished child for a duration of 2–3 months^(21)^.

The objective of the present study is to develop and formulate a nutrient dense ready to eat chicken liver–beef balls to end hidden hunger among pre- and school aged children, with particular emphasis on improving their growth and cognitive outcomes.

## Materials and methods

Ingredients of the liver meat recipe were purchased from the retail market and prepared in a batch of 110 kg. They included chicken liver, bovine meat, rice flour, together with other vegetables. The chicken liver was heated gently until water drainage ceased. The edible parts of carrots, garlic, onions and tomato were obtained by peeling followed by appropriate washing with tap water. The eight ingredients (Table 1) were homogenised electrically in a meat grinder and the resulting dough was shaped into round balls about 3 cm in diameter and fried in deep oil. The fried liver meat balls (LMB) were weighed, packed in paper bags and stored refrigerated for subsequent use.

**Table 1:** Ingredients used for preparing ready to eat liver meat balls

Ingredients	Quantity, kg	Price LE/kg	Total price LE
Meat	45	65	2925
Chicken liver	30	19	570
Onion	2·0	5	10
Garlic	2·5	15	37·5
Carrot	1·5	5	7·5
Tomato	2·5	3	12
Parsley	1·0	20	20
Rice flour	10	10	100
Oil	20	15	300
Weight, raw	114·5		3982
Weight, fried	99·2		
Number of balls = 1890 balls with average ball weight of 50 g.			2·1 LE/ball

LE, Egyptian pounds.

### Participating children

Preschool children aging 36–71 months were recruited from a child care centre (Kinder Garten) and children aging 73–118 months were recruited from a primary school located in urban Giza governorate.

Criteria for inclusion was the absence of gastro-intestinal disorders, no use of antibiotics during the last 3 months prior to the study; the taste of the LMB was acceptable to the child; the mother was willing to complete the dietary assessment sheet and to collect stool and urine samples from her own child at predetermined dates.

### Design of the study

The dietary intervention trial consisted of randomised controlled trial on sixty children. The children were assigned randomly into two age-matched equal groups: group 1 (36–< 72 months) and group 2 (≥ 72 months). Each group was subdivided into two subgroups: the first served as control and did not receive any supplement; while the second subgroup had a serving of LMB. The serving size was one LMB ball to children < 72 months; while the respective service size was two LMB balls for children ≥ 72 months. Serving was three times a week on Sundays, Tuesdays and Thursdays with a total of forty-five servings throughout the 3 months (90 d) duration of the trial.

### Anthropometric measurements

The age and sex of the child were obtained from the birth certificate; body weight and standing height measurements were taken, while the child was with the light clothes and bare footed. Based on the data on age, sex, body weight and height for each child, the respective three nutrition indices of physical growth: weight-for-age Z score, height-for-age Z score (HAZ) and weight-for-height Z score were computed by the EPIINFO software program. The child whose weight-for-age Z score measures below (–2·0) was underweight for his age. The HAZ index identifies linear growth and the child whose HAZ was below (–2·0) from the WHO child growth standards reference population was considered short for his age or stunted. The weight-for-height Z score measures body mass in relation to body length and the child whose weight-for-height Z score below (–2·0) was thin for his height^(22)^.

The BMR was computed in mega joules (MJ) using the following equations^(23)^. Boys 3–10 years = 0·095 ×body = weight + 2·11.

Girls 3–10 years = 0·085 × body weight + 2·03.The BMR was expressed in kcal from the relation 1 MJ = 4180 kcal.

### Dietary intake assessment

Three 24-h dietary recalls were collected on non-consecutive days from the mother of each child. The mothers recalled all foods and beverages consumed at home and at the day care centre or school throughout the previous 24 h (beginning from morning meal on the day before up to the morning meal on the day of the interview). All mothers were then asked to estimate the quantities of the mentioned foods and to state the ingredients used for preparing the dishes and their respective quantities. To facilitate the quantity estimates, standardised household measures (cups, plates and bowels) were provided by the interviewer and marked with several levels to indicate different contents. Raw ingredients were converted from household measures to weight estimates by taking reference weights, calculated on the basis of the average of weights on a digital weighing scale. A trained dietician instructed parents, checked the data and supervised inconsistencies and completeness of the records and was responsible for coding and computer entry of the consumption data. A raw data set for each child contained records with food codes and day, meal, quantity (g or ml), food item and its food code. The food intake data were converted into weights and the combined data were converted into respective energy and estimated nutrient intakes using the data values from the precise Egyptian food composition table (unpublished data) and other food data bases as needed^(19)^. Calculation of nutrient intake and food group consumption was performed using a software application, specifically developed for processing of dietary data.

To determine the adequacy of daily nutrient intake by a sample individual, the child’s daily nutrient intake was compared with the respective age and sex-specific FAO/WHO essential minerals and vitamins^(20)^ for expressing the individual daily intake of a nutrient into the respective probability of nutrient adequacy (PA). The percentage of reference for each nutrient was capped at 100 % daily value to avoid overvaluing by single nutrient.

#### Validation test

Ratios of estimated daily energy intake (mjoules/ BMR MJ) below 0·70 or above 1·80 were excluded from the calculation and the dietary assessment was repeated.

### Cognitive function

Wechsler Intelligence Scale for 3–5 years (grades Kinder Garten) through 10 years was carried out according to the standard methodology using the detailed instructions^(21)^. The test identifies how a child’s performance compares to age or grademates. It identifies specific skill strengths and deficits. A battery of cognitive verbal (three subtests) and performance or practical subtests were used. The verbal subtests included word reading, reading comprehension, oral listening, oral expression such as question answering and story generation, summarisation, attention difficulties and concentration difficulties or comprehension of written and spoken language and word fluency. Skill measures included picture completion; the child was asked to find the missing parts in a picture of familiar objects and people and the test is said to reflect visual alertness as well as visual recognition and identification (long-term visual memory). Age-based normative data was used and the total raw score from each of the subtests was transferred to the respective standard score for each subtest to obtain a weighted raw score. The composite standard scores were calculated by summing the subtest standard scores.

### Measurements and sample collection

Chemical analysis of the LMB moisture content, protein, fat and ash was determined according to the Association of Official Agricultural Chemists method^(22)^. Moisture was determined by heating the samples in an oven at 102°C until a constant weight was attained. The protein level was determined using the Kjeldahl method for determining nitrogen content and multiplying this by 6·25. After acid hydrolysis, the fat was extracted with ether using a Soxhlet apparatus. Ash was obtained by incineration at 500–550°C until the ash was carbon-free. The carbohydrate content was determined by difference. The energy density was calculated by multiplying the protein and total carbohydrate content by 16·736 kJ and adding the result to the fat content multiplied by 37·656 kJ.

### Laboratory investigations

#### Determination of blood Hb

Blood samples were collected at baseline (day 0) and endpoint (day 90) by a finger prick. Blood Hb concentration was measured in 20 µl whole blood by the direct cyanmethemoglobin method (Drabkin’s solution) and a photometer at 540 nm (Table 2).

**Table 2:** Hb concentrations (g/l) used to diagnose anaemia^(23)^

Population	Non-anaemic	Anaemic		
		Mild	Moderate	Severe
Children 6–59 months of age	110 or higher	100–109	70–99	< 70
Children 5–11 years of age	115 or higher	110–114	80–109	< 80

#### Assay of faecal Ig-A

At baseline (before the beginning of the randomised controlled trial) and at endpoint (day 22), each child was asked to collect stool sample into a container and an aliquot was immediately frozen at –20°C for later immunological assay.

The (faecal s-IgA) is the major Ig synthesised in the colonic mucous membrane and plays a major role in preventing adherence of pathogenic microorganisms to mucosal sites, and protects against gastrointestinal infections. The s-IGA was assayed by the Chrom ELISA kit (Bio Vender research Diagnostics, Cat # RIC6100, Germany) and was used according to the instruction of the manufacturer. Weighed faecal portions (80–120 mg) were suspended in 10-fold excess (1·5 ml) physiological phosphate buffer, vortexed and centrifuged at 13 000 rpm for 5 min. The supernatant was diluted 1:250 in wash buffer. Standards, controls and stool samples were simultaneously run in the ELISA and the concentration of s-IgA was read at 405 nm in ELISA reader against a standard curve and the results were expressed as mg faecal S-IgA per gram stool.

#### Urine analysis

Each child was asked after evacuating morning urine to collect void urine sample, which was labelled and saved frozen for subsequent laboratory investigations.

#### Analysis of urinary total hydroxyproline excretion

Hydroxyproline (HPRO) is a collagen-related marker as it is present in all fibrillar collagens and tissue breakdown contributing to urinary HPRO. Urinary hydroxyproline index (HPRO-I) is a potential biomarker with high prognostic value before growth spurt with cut-off point of ≥ 2·5 ± 0·9 among normal children. This biomarker assists in making the prospect of catch-up growth rate over short periods of time and in close monitoring of treatment regimens.

The determination of total urinary (U-HPRO) is based on the colorimetric reaction of Ehrlich^(24)^. Urine aliquots (100 µl) were hydrolysed in 10 × 1·5 cm pyrex tubes with screw caps with equal volume of 12 normal hydrochloric acid by heating at 125°C for 2 h. Upon cooling, the pH of the acid hydrolysate was adjusted to 6·0 by the addition of buffer solution. The standard hydroxyproline, the blank and the urine samples were subjected to colour reaction, which is based on the formation of pyrole derivative with tosylchloramine. The pink colour developed with p–aminobenzaldehyde was read at 560 nm, and the results were expressed as μg total HPRO per ml urine.

#### Urinary creatinine excretion

The classical alkaline picric acid and sodium hydroxide reagent were used^(25)^.

The U-HPRO excretion was expressed as total hydroxyproline/creatinine(mmol/mol). The urinary hydroxyproline index (U-HPRO-I) = U-HPRO creatinine ratio/kg body weight^(26)^.

#### Measuring of urinary iodine

Measurement of iodine concentrations from casual urine is now widely accepted as the best and most cost-effective way of monitoring iodine deficiency in a community, with an iodide excretion below the cut-off point of 0·79 µM iodine/l (100 µg/l) of urine is considered to be at risk of a marginal iodine supply and therefore of goitre and other iodine deficiency disorders. The determination of urinary iodine by ICCIDD^(27)^ urine aliquots (250 µl) was digested in 10 × 1·5 cm pyrex tubes with screw caps with ammonium persulfate in a heating block at 100° for 60 min. The catalytic properties of the liberated iodine were used for the reduction of ceric ammonium sulphate in the presence of arsenious acid, and the yellow colour was measured at 430 nm. A standard curve prepared from potassium iodate solution (10–80 ppm) was used for calculating the urinary iodine. Quality control for iodine was maintained by including reagent blanks to monitor contamination and estimate detection limits.

#### Determination of urinary thiobarbituric acid

Urinary malondialdehyde excretion is a valid biomarker for the oxidative stress of the body to lipid peroxidation, which is assayed by the thiobarbituric acid reactive species (TBARS). Colorimetric method^(28)^ and the results were expressed as urinary thiobarbituric acid creatinine ratio (mg/g).

### Statistical analysis

Descriptive statistics were obtained for all variables and outcome variables stratified by age groups: < 72 and ≥ 72 months. The Whisker box plots of each variable were classified according to age group and dietary treatment. The boxes indicate 25th to 75th percentiles, with mean relative abundances marked as lines and whiskers indicating the range (minimum/maximum) from the boxes. Statistical tests for the distribution of the variable were calculated and compared according to categories based on the Kruskal–Wallis test for continuous variables. Spearman correlation coefficients were used to measure the correlation.

## Results

### The composition of the liver meat balls supplement

Table 3 presents the composition of the macronutrients and selected minerals, trace elements and vitamins of the LMB.

**Table 3:** Composition of liver meat balls (LMB) and contribution to the daily diet (3 d/week)

Ingredients	LMB composition		Per LMB child per day	
	Two balls	One ball	< 72 months	≥ 72 months
Quantities, g	100	50	21·4	43
Protein, g	17·64	8·82	3·78	7·56
Animal protein	16·62	8·31	3·56	7·12
Fat, g	25·74	12·87	5·51	11·03
Energy, kJ	1388	694	297·4	594·8
Fe, mg	4·34	2·17	0·93	1·86
Fe, animal	4·16	2·08	0·89	1·782
Zn, mg	3·21	1·60	0·69	1·37
Se, µg	21·66	10·83	4·64	9·28
Vitamin A, RE	7300	3650	1564·29	3128·57
Vitamin E, mg	0·33	0·17	0·07	0·14
Vitamin B_1_, mg	0·24	0·13	0·05	0·10
Vitamin B_2_, mg	0·86	0·43	0·18	0·37
Vitamin B_6_, mg	0·65	0·32	0·14	0·28
Vitamin B_12_, µg	12·79	6·39	2·74	5·48
Folate, µg	43·77	21·89	9·38	18·76
Niacin, mg	10·66	5·33	2·28	4·57

### Characteristics of participants at baseline and after supplementation with liver meat balls

**Fig. 1:**
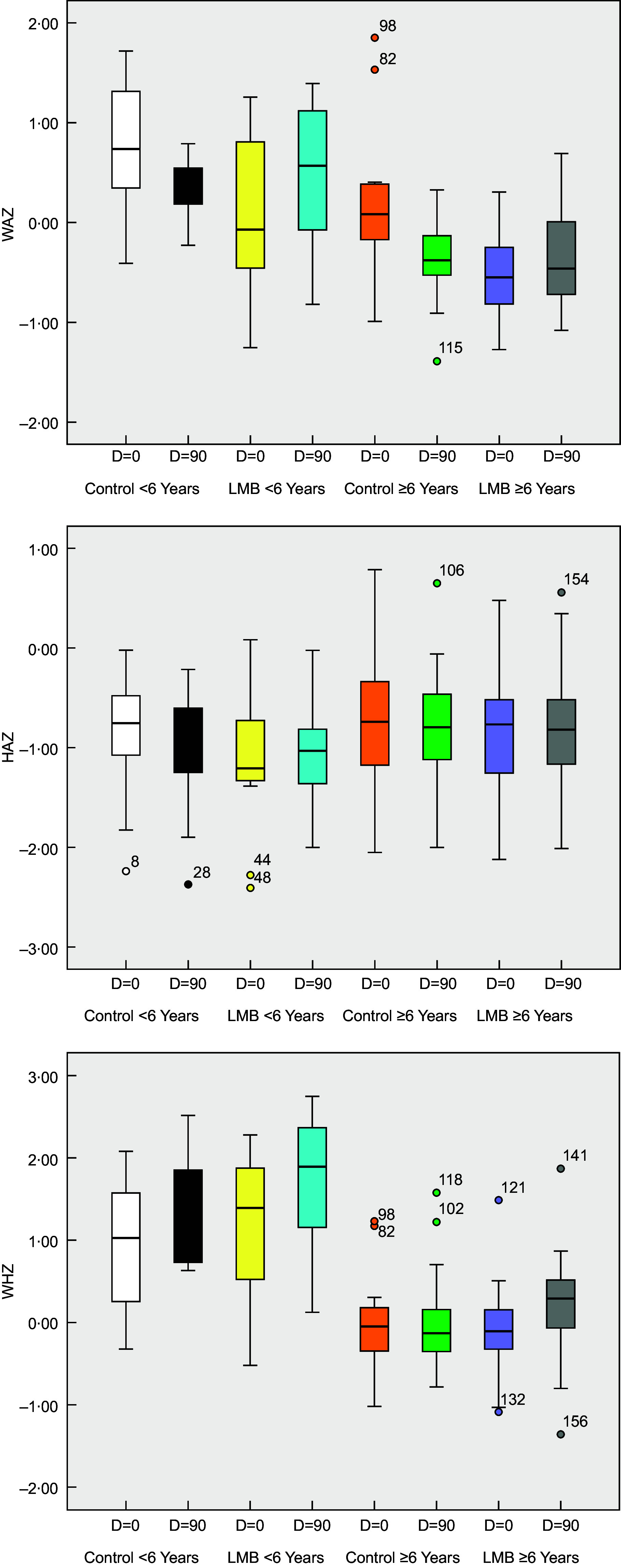
Whisker box plot for the three growth indices among control and LMB children belonging to two age groups at baseline and at 90 d post-intervention

Table 4 and Fig. 1 presents the characteristic of the participating children classified according to age group and dietary intervention. The results of anthropometric assessment show that the overall mean baseline values of weight-for-age Z score and HAZ of the control and LMB group were within the normal range and above –2·0. Prevalence of stunting was 7·7 % % (HAZ < –2·0) among the control < 72 months. None of the children in the LMB group had HAZ – < 2·0.

**Table 4:** Baseline characteristics of the participating children and the effect of the 3-month intervention with LMB

Age group	Control group								LMB supplement							
	Children < 72 months				Children ≥ 72 months				Children < 72 months				Children ≥ 72 months			
	Day 0		Day 90		Day 0		Day 90		Day 0		Day 90		Day 0		Day 90	
	Mean	se	Mean	se	Mean	se	Mean	se	Mean	se	Mean	se	Mean	se	Mean	se
Number	13		13		17		17		13		13		17		17	
Age, months	57·82	2·84	61·2	2·65	95·6	3·04	98·6	3·04	52·9	3·59	55·9	3·59	92·8	2·92	95·8	2·92
WAZ	0·71	0·18	0·27	0·09	0·18	0·16	−0·37	0·11	0·07	0·22	0·48	0·19	−0·59	0·1	−0·39	0·1
WAZ days 0–90			−0·44	0·17^a^			−0·56	0·14^a^			0·41	0·06^c^			0·19	0·04^b^
HAZ	−0·73	0·21	−0·96	0·18	−0·79	0·17	−0·88	0·16	−1·11	0·19	−1·07	0·14	−0·01	0·05	−0·79	0·16
HAZ, days 0–90			−0·23	0·1			−0·09	0·03			0·03	0·08				
WHZ	0·85	0·21	1·24	0·22	0·00	0·14	0·06	0·15	1·08	0·26	1·66	0·23	−0·17	0·11	0·10	0·13
WHZ, days 0–90			0·38	0·10			0·06	0·10			0·58	0·08			0·27	0·06
Blood Hb, g/dl	11·4	0·20^a^	11·47	0·20^a^	11·55	0·12^a^	11·67	0·13^a^	11·6	0·19^a^	12·9	0·15^b^	11·66	0·12^a^	12·8	0·12^b^
Non-anaemic, %	23		23		17·65		23·53		30·77		100		23·53		88·23	
Moderate anaemic, %	30·77		30·77		17·65		0		15·38		0		5·88		0	
Mild anaemic, %	46·15		46·15		64·70		76·47		53·85		0		70·59		11·76	
Syst IgA, mg/g stool	2·9	0·13	3·13	0·18	3·17	0·17	3·00	0·18	2·92	0·20	8·72	0·31	3·33	0·2	7·89	0·47
Urin-HPRO-I	3·27	0·27	3·09	0·24	3·09	0·19	3·13	0·18	2·48	0·25	5·97	0·4	1·77	0·09	4·30	0·17
U-HPRO-I, < 2·5, %	23·1		38·5		23·5		23·5		53·8		0		94		0	
Urin-I, µg/l	154·26	5·32	148·53	6·69	162·04	5·14	160·60	6·25	170·12	5·09	204·55	4·26	146·47	4·22	187·36	5·41
Urin-I, < 100 µg/l	0		0		0		0		0		0		0		0	
TBA	45·35	2·77	43·05	2·64	52·01	3·01	53·04	3·11	39·65	4·25	37·01	3·79	36·64	1·83	31·70	1·49

LMB, liver meat ball; WAZ, weight-for-age Z score; HAZ, height-for-age Z score; WHZ, weight-for-height Z score; HPRO, hydroxyproline; TBA, thiobarbituric acid.

Within the same row, mean values are significantly different (*P* < 0·05), if they do not share the same alphabet.

### Prevalence of anaemia at baseline and after liver meat balls supplementation

**Fig. 2:**
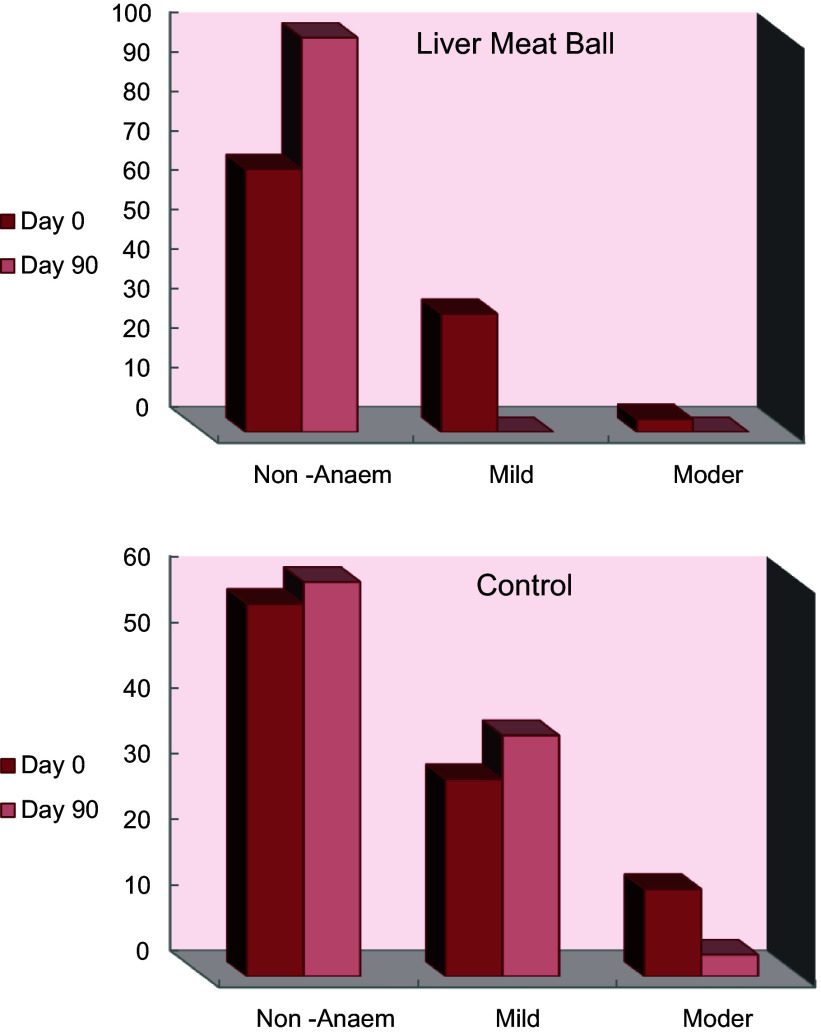
Prevalence of non-anaemic, mild and moderate anaemia among control and LMB children at days 0 and 90

Initially, 43 % of all participating children were suffering from mild or moderate anaemia. Following the intake of the LMB supplement for 90 d, anaemia disappeared completely among children in the age group < 72 months, and among the older children ≥ 72 months its prevalence was reduced down to 12 %; while its prevalence remained almost unchanged among the control group (Fig. 2). The mean increases in the concentration of blood Hb (Δ d90–d0) were 1·3 ± 0·08 and 1·15 ± 0·09 g/dl among children < 72 months and ≥ 72 months, respectively. Chi-square test (χ^2^) revealed high statistical significant differences (*P* < 0·001) between the control and the LMB group in the distribution of the number of anaemic and non-anaemic children.

### Faecal systemic Ig A at baseline and after liver meat balls supplementation

**Fig. 3:**
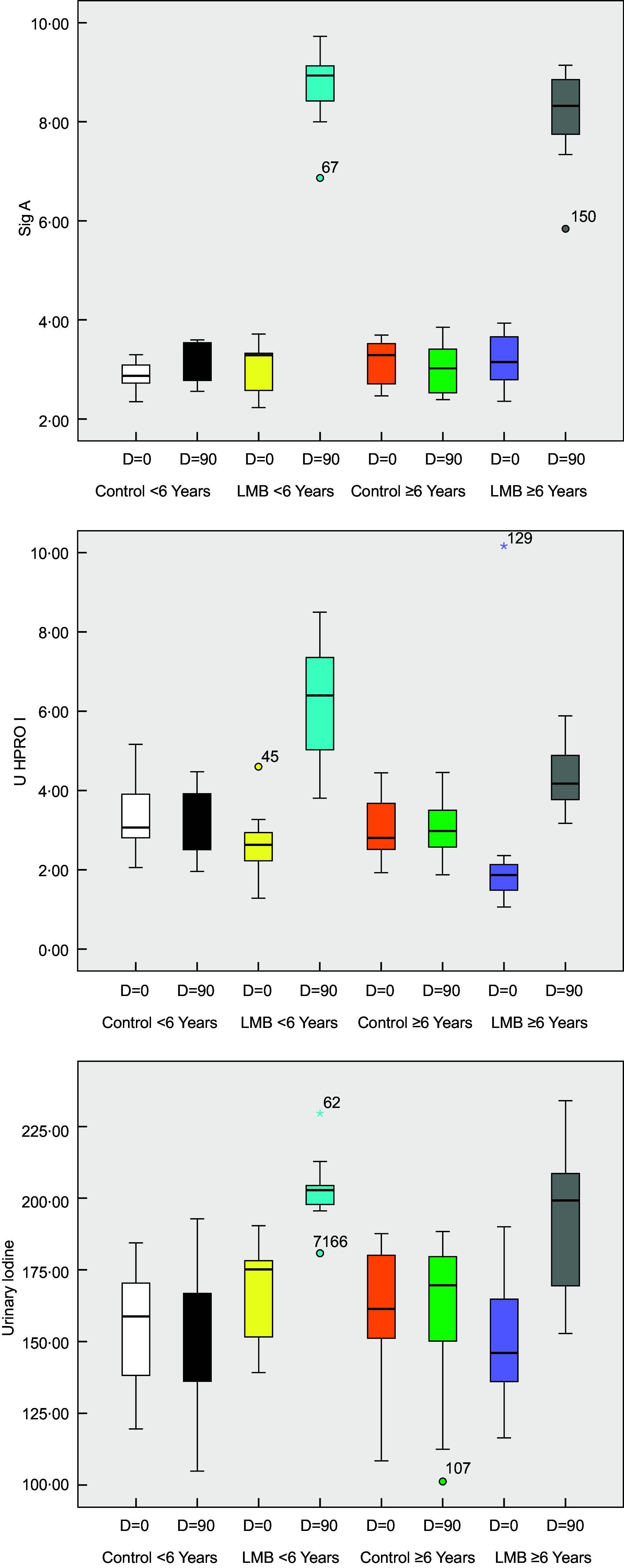
Whisker box plots for faecal systemic IgA, urinary H-PRO-I and urinary Iodine among control and LMB children belonging to two age groups at baseline and at 90 d post-intervention

The baseline faecal concentration of faecal systemic Ig A (s-Ig A) averaged 3·1 ± 0·1 µg (2·87–3·68) per gram faecal material with no significant differences between the groups. The 90-d dietary intervention with LMB was associated with a significant increase (*P* < 0·05) in the mean concentration of s-Ig A compared with the respective pre-intervention levels and compared with the control group (Table 4 and Fig. 3).

### Urinary hydroxyproline index at baseline and after liver meat balls supplementation

At baseline, the mean (U-HPRO-I) excretion was significantly higher (*P* < 0·05) among children < 72 (4·08 ±0·17) compared to those aging ≥ 72 months (2·39 ± 0·12). The prevalences of children excreting U-HPRO-I) < 2·5 (the cut-off point for normal biochemical growth) amounted to 28·2 % and 63% among children < 72 months and ≥ 72 months, respectively. Following the 90-d dietary intervention with LMB, the excretion of U-HPRO-I exceeded the cut-off point of 2·5; while the respective excretions of U-HPRO-I below 2·5 were 66 % and 82·4 % among the control group aging < 72 and ≥ 72 months (Table 4 and Fig. 3). Chi-square test (2 × 2 table) revealed very high statistical significance (*P* < 0·001) between the control and the LMB children in the distribution of numbers excreting U-HPRO-I below 2·5 and ≥ 2·5 compared with the expected based on normal distribution.

### Urinary iodine excretion at baseline and after liver meat balls supplementation

At baseline, urinary iodine excretion of all children was above 100 µg, the cut-off point of iodine deficiency, reflecting adequate Iodine intake. It is noteworthy that the salt iodination program is effective in Egypt since 1996, which clearly has positive implication on the iodine status of the vulnerable population.

Following the 90-d intervention with LMB, those children < 72 and ≥ 72 months of age excreted in their urine on average 20 % and 28 % higher iodine (*P* < 0·05) compared with the respective pre-intervention values (Table 4 and Fig. 3) . The control group did not show such changes though the estimated daily iodine intakes were almost comparable. This is more likely due to lesser iodine excretion in the faeces among the LMB group, because it is well known that vegetarians are at high risk of developing iodine deficiency.

### Urinary excretion of thiobarbituric acid at baseline and after liver meat balls supplementation

The mean urinary excretion of thiobarbituric acid was not affected by the intervention with LMB and the differences in concentrations between all groups did not attain significant levels (*P* > 0·05).

### Estimated daily nutrient intakes at baseline and after liver meat balls supplementation

Tables 5 and 6 present the estimated daily intakes of energy, six macronutrients (protein, fat, Na, potassium, Ca and Mg) and thirteen micronutrients (Fe, Zn, Se, iodine, vitamins A, E, B_1_, B_2_, B_6_, B_12_, folate, niacin and vitamin C among children classified according to age group < 72 and ≥ 72 months, respectively. At baseline, there were no significant differences in the estimated daily intakes between the control and the LMB groups except for higher vitamin C intake (*P* < 0·05) among the control children (32·8 ± 3·2 mg) compared with the respective intakes of 16·7 mg among the LMB children. The estimated daily intake of Fe increased significantly (*P* < 0·05) from 4·65 ± 0·25 mg on pre-intervention days up to 5·5 ± 0·23 mg during the intervention days with LMB (days 1–90) days. Similar significant increases were found in the estimated daily vitamin A intake following the dietary intervention with LMB (days 1–90) compared with the pre-intervention intake.

**Table 5:** Estimated daily intakes of energy and essential nutrients among children below 72 months of age classified according to dietary intervention

Energy/nutrient			Liver meat balls (LMB) group			
	Control group		Pre-intervention		Intervention^*^	
	Mean	se	Mean	se	Mean	se
Food intake, g	646·8	30·2	550·1	26·1	571·5	26·1
Energy, kJ	4233	272	851·0	149	3857·65	149
Energy intake/BMR	1·0	0·1^a^	1·0	0·04^a^	1·2	0·04^b^
Protein, g	39·1	2·28	34·5	2·2	38·3	2·2
Protein, animal %	59·4	3·0	57·2	2·6	60·7	2·6
Protein %E	15·9	1·0	16·1	0·57	16·1	0·57
Fat, g	37·8	3·1	32·5	1·55	38·0	1·55
Fat, % E	34·1	1·57	34·6	1·44	34·6	1·44
Na, mg	1090·3	91·6	949·4	60·2	949·4	60·2
Potassium, mg	4499·1	725·1	2419	522	2419·5	522·2
Ca, mg	507·2	30·9	523·2	24·1	523·2	24·14
Mg, mg	142·8	14·8	106·8	4·45	106·8	4·45
Fe, mg	5·3	0·46^a,b^	4·65	0·25^a^	5·5	0·23^b^
Fe, FMP, % total^**^	24·75	3·4	22·5	3·19	23·4	3·19
Zn, mg	5·27	0·41	6·3	0·59	7·0	0·6
Se, µg	20·88	3·28	17·66	1·16	21·28	1·2
Iodine, µg	82·3	7·5	89·5	9·3	87·9	10·0
Vitamin A, RE	132·8	24·0^a^	498·0	252·8^a^	2062	252·8^b^
Vitamin E, mg	2·06	0·29	1·20	0·22	1·27	0·22
Thiamine, mg	0·65	0·13	0·74	0·12	0·79	0·12
Riboflavin, mg	0·78	0·14	0·92	0·13	1·07	0·13
Vitamin B_6_, mg	1·0	0·20	1·15	0·19	1·29	0·19
Vitamin B_12_, µg	2·23	0·18^a^	3·74	1·4^a,b^	6·48	1·41^b^
Folate, µg	89·75	7·4	92·4	6·4	101·76	0·77
Niacin, mg	15·6	0·9	13·7	0·77	15·9	6·4
Vitamin C, mg	32·8	3·2^b^	16·7	2·8^a^	16·66	2·8^a^

* Intervention = days 1–90 intakes of one (LMB) ball three times per week.

** Fe, FMP = iron derived from fish, meat and poultry.

Mean values are significantly different (*P* < 0·05), if within the same row they do not share the same alphabet (Student’s *t* test).

**Table 6:** Estimated daily intakes of energy and essential nutrients among children above 72 months of age classified according to dietary intervention

Energy/nutrient			Liver meat balls (LMB) group			
	Control group		Pre-intervention		Intervention^*^	
	Mean	se	Mean	se	Mean	se
Food intake, g	851	28·5^b^	614·1	27·9^a^	657	27·9^a^
Energy, kJ	5924·5	225·9^b^	4585·7	194·6^a^	5179·8	194·6^a,b^
Energy intake/BMR	1·0	0·04^a^	1·13	0·04^a^	1·47	0·06^b^
Protein, g	50·7	2·9	43·3	2·49	50·9	2·49
Protein, animal %	49·6	2·5^a^	57·0	2·3^a,b^	63·1	1·97^b^
Protein %E	14·0	0·7	15·8	0·54	16·3	3·3
Fat, g	54·5	3·2	47·6	2·5	56·3	3·6
Fat, % E	34·3	1·1^a^	39·0	1·2^b^	38·6	2·49^a,b^
Na, mg	1471	94	1224	143	1169	158
Potassium, mg	6132	629^b^	2335	430^a^	2348	427^a^
Ca, mg	663	36·3	557·5	33·7	578·5	33·8
Magnesium, mg	184	13·9	145·6	9·8	158·8	17·5
Fe, mg	6·78	0·35^a,b^	6·14	0·36^a^	8·0	0·36^b^
Fe, FMP, % total^**^	18·3	2·5^a^	35·5	5·6^b^	35·5	6·2^b^
Zn, mg	6·3	0·4	6·0	1	7·4	0·36
Se, µg	29·6	3·7	32·7	7·8	42·0	7·8
Iodine, µg	106	7·4	76·8	11·6	80·2	7·07
Vitamin A, RE	410	109^a^	646·4	214·7^a^	3774	214·7^b^
Vitamin E, mg	2·52	0·36	2·2	0·42	2·3	0·42
Thiamine, mg	1·26	0·25	0·8	0·11	0·9	0·11
Riboflavin, mg	1·63	0·25^b^	0·84	0·12^a^	1·20	0·12^a,b^
Vitamin B_6_, mg	1·66	0·19	1·29	0·20	1·57	0·20
Vitamin B_12_, µg	3·27	0·49^a^	4·15	1·17^a^	9·6	1·17^b^
Folate, µg	121·4	7·2	119	11·38	137·7	11·4
Niacin, mg	20·13	1·29	20·27	1·53	24·8	1·5
Vitamin C, mg	46·0	2·2^b^	21·7	3·02^a^	21·7	3·0^a^

BMR, basal metabolic rate; RE, retinol equivalent.

* Intervention = days 1–90 intakes of two (LMB) balls three times per week.

** Iron, FMP = Fe derived from fish, meat and poultry.

Mean values are significantly different (*P* < 0·05), if within the same row they do not share the same alphabet (Student’s *t* test).

With regard to the children aging ≥ 72 months, higher estimated daily intakes (*P* < 0·05) were found in the energy among the control children (1416 ± 54 kcal) compared with the respective intake of 1096 ± 46·5 kcal among the LMB children (Table 6). The estimated dietary intake of protein was adequate in both groups, and roughly half the dietary proteins were derived from animal sources. Dietary fat made up 39·0 ± 1·2 % of the total energy in the diet of the LMB children and was significantly higher (*P* < 0·05) than the respective fat % Energy of 34·3 ± 1·1 % E in the diet of the control children. The estimated Fe intake was almost comparable (6·14–6·78 mg/d) among both groups; but 35·5 % of the dietary Fe in the diet of the LMB children was derived from the animal sources; fishes, meats and poultry *v*. only 18·3 % among the control. On the contrary, the estimated dietary vitamin C supply in the diets of control children averaged 46 ± 2·2 mg; which was double the respective supply of 21·7 ± 3·02 mg among the LMB children. Dietary vitamin E was poor in the diets of all children.

### The probability of dietary adequacy at baseline and after liver meat balls

The probability of adequacy (PA) of thirteen nutrients is presented in Tables 7 and 8. The intervention with LMB (days 1–90) was associated with significant increases (*P* < 0·05) in the PA of total Fe, Fe from animal sources (fishes, meats and poultry) and vitamin A. Increases were also observed in the PA of Zn, Se, vitamins B1, B2, B6, folic acid and niacin, but the increase did not attain significant level (*P* > 0·05).

**Table 7:** Probability of adequacy of the diet of control and LMB children < 72 months at pre-intervention and during the intervention period (days 1–90)

Nutrient			LMB group			
	Control		Pre-intervention		Intervention^*^	
	Mean	se	Mean	se	Mean	se
Ca	87·9	4·9	91·3	2·41	88·7	3·0
Mg	100	0·0	100	0·0	100	0·0
Fe	85·7	4·6^a^	81·16	4·39^a^	100	0·0^b^
Zn	84·7	5·9	95·36	2·81	97·8	1·6
Vitamin A	29·4	5·43^a^	42·3	7·64^a^	100	0·0^b^
Vitamin E	41·1	5·71	24·1	4·38	25·3	4·4
Vitamin B_1_	83·2	5·0	88·2	4·87	92·2	3·9
Vitamin B_2_	91·9	3·3^a^	95·58	3·2^a,b^	99·4	0·61^b^
Vitamin B_6_	94·1	3·51	94·8	2·85	99·4	0·43
Vitamin B_12_	100	0·0	100	0·0	100	0·0
Folic acid	46·9	3·87	50·29	2·53	53·0	2·9
Niacin	100	0·0	100	0·0	100	0·0
Vitamin C	91·2	6·01^b^	53·5	8·48^a^	53·4	8·47 ^a^
Average PA	79·5	6·66	78·2	5·33	85·3	6·87

LMB, liver meat balls; PA, probability of adequacy.

Mean values are significantly different (*P* < 0·05), if within the same row they do not share the same alphabet (Student’s *t* test).

* Intervention = days 1–90 intakes of one liver meat ball three times per week.

**Table 8:** Probability of adequacy for thirteen essential nutrients in the diets of children ≥ 72 months classified according to the dietary intervention

Nutrient			Liver meat ball supplement			
	Control group		Pre-intervention		Intervention	
	Mean	se	Mean	se	Mean	se
Ca	90·4	3·34	79·4	3·9	79·4	3·9
Mg	100	0	99·4	0·55	99·4	0·56
Fe	84·2	4·75^a^	81·1	5·0^a^	97·8	1·0^b^
Zn	92·5	3·35	88·9	4·1	97·6	2·35
Vitamin A	58·7	7·34^a^	59·7	7·57^a^	100^b^	
Vitamin E	36·6	5·0	30·4	5·3	32·3	5·2
Vitamin B_1_	85·3	4·1	75·3	5·2	82·6	4·1
Vitamin B_2_	95·0	2·5^b^	73·3	6·38^a^	94·6	2·4^b^
Vitamin B_6_	95·2	3·12^a,b^	83·3	4·0^a,b^	97·2	1·3^b^
Vitamin B_12_	96·8	2·58	92·9	4·9	100	
Folic acid	41·7	2·74	42·3	3·7	49·1	3·76
Niacin	98·9	1·13	100		100	
Vitamin C	98·9	1·13^b^	61·0	8·0^a^	61·0	8·0^a^
APA %	82·6	19·58	74·4	5·85	78·9	6·26

APA, American Psychological Association.

Within the same row, mean values are significantly different (*P* < 0·05), if they do not share the same alphabet.

### Cognitive function by Wechsler test at baseline and after LMB

Scaled scores of the verbal and non-verbal cognitive function (Table 9). The changes in the scaled score (Δ day 90–day 0) of each subtest among the control and LMB children are presented in Table 9. Several of these scaled score increased significantly following the 90 d intervention with LMB and the degree of statistical significance was age-dependent. The digit span test was the only subtest which did not increase following the 90 d intervention with LMB. It is a measure of attention, short-term auditory memory and auditory sequencing (Table 9). Changes (Δ d90–d0) of a number of verbal and non-verbal subtests correlated positively and significantly (*P* < 0·05) with the increases (Δ d90–d0) in absolute body heights, the HAZ and urinary iodine excretion. In children ≥ 72 months, the increase in practical subtests of the Wechsler score correlated with changes in HAZ (Δ d90–d0) (Table 10).

**Table 9:** Effect of dietary intervention with liver meat balls (LMB) on Wechsler intelligence scale for children classified according to age group (Mean ± se)

	Children below 72 months of age									Children ≥ than 72 months								
	LMB supplement				Control group					LMB supplement				Control group				
	Pre		Post		Pre		Post			Pre		Post		Pre		Post		
Scale	Mean	se	Mean	se	Mean	se	Mean	se	*t* test, P	Mean	se	Mean	se	Mean	se	Mean	se	*t* test, P
Information	9·3	0·54	10·2	0·44	9·3	0·33	9·2	0·43	1·49	9·2	0·43	10·2	0·34	9·5	0·52	8·9	0·42	2·22^*^
Comprehension	10·1	0·39	11·0	0·32	10·1	0·37	10·0	0·42	2·62^*^	8·2	.38	9·3	0·37	7·7	0·35	7·8	0·32	1·888^*^
Arithmetic	9·5	0·45	11·0	0·38	9·5	0·45	11·0	0·38	2·55^*^									
Digit span										7·1	0·52	8·2	0·62	6·8	0·44	6·8	0·52	1·374
Verbal tests, SS	28·9	1·0	32·2	0·65	28·5	0·72	28·5	0·83	3·03^**^	24·6	0·44	27·8	0·57	24·0	0·74	23·5	0·6	3·53^**^
Full SS	57·8	2·02	64·5	1·30	57·1	1·44	57·1	1·66	3·0^**^	49·2	0·88	55·5	1·13	48·0	1·48	47·1	1·2	3·53^**^
IQ	105·0	2·25	111·9	1·35	104·2	1·74	104·4	1·95	2·936^**^	99·0	1·12	107·5	1·44	97·3	1·91	96·2	1·5	3·72^**^
Picture complete SS	8·1	0·63	9·4	0·64	7·7	0·48	7·5	0·46	3·27^**^	6·2	0·47	7·1	0·47	6·3	0·47	6·3	0·54	1·887^*^
Animal house, SS	11·1	0·56	12·1	0·60	11·1	0·52	11·3	0·51	4·26^***^									
Coding										9·0	0·15	10·3	0·27	9·5	0·40	9·6	0·41	2·224^*^
Maze, SS	5·77	0·57	6·6	0·56	5·2	0·44	5·4	0·47	1·81^*^	8·0	0·51	9·8	0·71	8·1	0·53	8·3	0·45	1·73
Practical, SS	25·0	1·58	28·1	1·66	24·0	1·25	24·1	1·23	5·2^***^	23·2	0·55	27·2	0·63	23·9	0·70	24·3	0·6	3·147^**^
Practical, Full SS	50·0	3·16	56·3	3·31	48·0	2·49	48·3	2·47	5·2^***^	46·5	1·10	54·3	1·25	47·9	1·41	48·7	1·2	3·147^**^
Practical, IQ	78·9	5·62	95·8	3·42	76·5	4·81	87·0	2·48	1·13	94·9	1·55	106·1	1·74	97·1	2·0	98·1	1·7	3·26^**^
Total cognitive, SS	53·9	1·94	60·4	1·96	52·5	1·33	52·7	1·45	5·16^***^	47·8	0·45	54·9	0·63	47·9	0·60	47·9	0·5	13·0^***^
Total cognitive, Full SS	107·8	3·88	120·8	3·9	105·1	2·66	105·4	2·9	5·16^***^	95·6	0·90	109·9	1·25	95·9	1·21	95·8	0·9	6·52^***^
Total cognitive, IQ	97·3	2·01	104·1	2·03	80·1	5·76	95·6	1·50	4·85^***^	96·9	0·63	107·2	0·92	97·1	0·9	96·9	0·7	6·50^***^

SS, scaled scores.

Within the same row, and among each age category, mean values are significantly different, if they do not share the same asterisk.

* *P* < 0·05.

** *P* < 0·01.

*** *P* < 0·001.

**Table 10:** Simple correlation coefficients (Pearson *r*) between verbal and non-verbal subtests and selected growth indices and biochemical indicators tests

Wechsler score
	Children < 72 months					Children ≥ 72 months				
	Height, cm	HAZ	Hb, g/l	U-I	TBA	Height, cm	HAZ	Hb, g/l	U-I	TBA
Full SS	0·163	0·163	0·241							−0·316^*^
IQ	0·147	0·157	0·262							−0·297
Arithmetic					−0·219					
Comprehension	0·163	0·163	0·241							
Digital span										−0·3485^*^
Information	0·279	0·304^*^	0·208	0·107		0·414^**^				−0·146
Animal house			0·195	0·333^*^						
Coding							0·349^*^			−0·285
Maze	0·409^**^	0·406^**^	0·118		−0·129	0·370^*^	0·349^*^	0·264		
Complete picture	0·121									−0·316^*^
Non-verbal total,		0·422^**^				0·235	0·203	0·240		
Full SS		0·422^**^				0·235	0·202	0·240		
Total cognitive function Full SS		0·373^*^						0·105		−0·264
IQ		0·373^*^				0·134	0·101	0·101		−0·316^*^

HAZ, height-for-age Z score; TBA, thiobarbituric acid; SS, scaled scores.

* *P* < 0·05.

** *P* < 0·01.

## Discussion

The development of the deep-fried LMB used in the present study was culturally acceptable by the participating children, with shelf-life stability for days, reducing the need for refrigeration and is part of the ASF best food-based strategy to prevent stunting and promote cognitive development.

Body weight contains an average of 70 ml blood/kg. Hb contains 3·4 mg Fe/g and the normal blood Hb concentration is 115–120 g/l^(30)^. It is estimated that if 30% of the population are anaemic at the onset, an appropriate theoretical sample size of about twenty-five subjects is needed to be followed for at least 3 months to detect an increase in Hb of 1 g/dl^(35)^. This protocol was practiced in the present study, and the sample size was thirty children and indeed at the end of the 90 d intervention with LMB, the increases in blood Hb concentration averaged 1·3 ± 0·08 and 1·15 ± 0·09 g/dl among children < 72 months and ≥ 72 months, respectively.

Fe is present in food in both inorganic (ferric and ferrous) and organic (mostly heme). Heme Fe constitutes 40 % of the Fe in meat, poultry, chicken liver and fish, whose absorption into the intestinal lumen is 25 % times greater^(36)^ than dietary non-heme Fe^(37)^. Full fat soy flour, isolated soy protein concentrate and even textured soybean are typical example of food products reducing Fe absorption by binding it to insoluble peptides in the duodenum and the term residual ‘soy factor’ is implicated^(38)^. Commercial ground beef patties and hamburger available on the Egyptian market contain soyproteinsas ingredient in the range between 10 and 25 %, as detected by immunological blotting technique^(39)^. A randomised controlled trial showed statistical significant (*P* < 0·05) inferior % apparent Fe retention among male adolescents consuming the sausage extended with soyabean protein (14·6 ± 0·8 %) compared with the respective % apparent Fe retention of 32·2 ± 1·9 % found among the group consuming Fe from 100 % beef under otherwise identical experimental conditions^(40)^. The consumers welcome the slight reduction in the price of meat extended with soyabean proteins, being unaware that this is at the expense of inferior qualities. % Fe bioavailability can be assessed precisely from the daily intakes of the food constituents of Fe from fish (F), meat (M) and poultry (P)^(41)^. The intake of high dietary ascorbic acids, such as guava (102 mg/100 g) overcome the inhibitory effects of phytate in cereals and enhanced significantly Fe bioavailability in adolescents boys and girls^(42)^.

Also, vitamin A is needed together with Fe for the most effective treatment of anaemia^(43)^. Vitamin A deficiency impairs the utilisation of Fe for haemoglobin synthesis, in part by trapping Fe in the liver and spleen^(44)^. Baseline dietary supply of vitamin A in the diets of our participating children was poor, and the intervention with the LMB supplement restored the deficiency and the probability of vitamin A adequacy exceeded 100 %. A South African study carried out on preschool children attributed the adequate vitamin A status of the children to regular intake of 60–75 g of liver once per month^(20)^. Riboflavin deficiency also impairs Fe absorption by altering the turnover of intestinal cells, associated with a change in Fe utilisation for Hb synthesis^(45)^. The estimated daily intake of riboflavin was acceptable among our children and was close to 90 % of the FAO/WHO requirement. Both vitamin B_12_ and folic acid deficiencies can cause anaemia. The intervention with LMB provided a generous supply of vitamin B_12_ averaging 2·74 and 5·48 µg/day, for children < 72 and ≥ 72 months, respectively, which exceed the respective daily FAO/WHO requirement.

Mild anaemia (Hb concentration between 110 and 119 g/l) was reported to affect the behaviour and cognitive functions of 9-year-old school children^(46)^. Fe supplementation of the anaemic children with high pharmacological dosage of 100 mg ferric glycine for 10 weeks increased the blood Hb concentration and the increase was also associated with statistically significant improvement in total The Wechsler Intelligence Scales for Children-Revised score^(46)^. Our results are in good agreement with these findings.

Another study reported that supplementing the basal diet of children from Bhurkino Faso, Ghana, Malawi^(17)^ and Kenya^(16)^ with small amounts of meats improved their linear growth, physical activity and increased their cognitive skills, behaviour and initiative. Eight interventional studies across eleven countries indicated that supplementation with ASF to 26 299 children (age 1–16 years) showed consistent positive associations with cognitive development, increased cognitive functions (test and exam scores) by up to 20-fold and fluid intelligence and verbal skills by up to twofold^(47)^.

Neurological studies showed that over a hundred unique factors have been identified to influence cognitive development in children^(48)^. The high-quality proteins found in ASF are typical examples in facilitating specific mechanisms, such as speed of information processing, that are involved in learning tasks such as problem-solving capacity^(18)^. The bioavailability of other nutrients in ASF such as Fe, Zn, iodine and B vitamins (B_12_, B_6_, folate and riboflavin) enhance cognitive development through their impact on structural brain development via enhancement of myelination and synaptic connectivity.

## Conclusion

The present study clearly demonstrates that Fe and vitamin A deficiencies are risk factors affecting children’s growth and cognitive development. Since venous blood sampling from healthy children is prohibited by law in Egypt, it was not possible to assess the biochemical status of vitamin A status and the dietary assessment was the only option to assess the estimated vitamin A intake. The study also clearly demonstrated the role of ASF against undernutrition conditions that generate low/suboptimal children’s cognitive development. Therefore, dietary intervention using ASF should be articulated, publicised and disseminated sufficiently^(49)^ in order to be the only sustainable long-term approach at the community level, where designing the most effective and practical strategies^(50)^.
